# Capturing the unpredictability of stem cells

**DOI:** 10.7554/eLife.95513

**Published:** 2024-03-01

**Authors:** Arda Durmaz, Valeria Visconte

**Affiliations:** 1 Department of Translational Hematology and Oncology Research, Taussig Cancer Institute, Cleveland Clinic Cleveland United States

**Keywords:** healthy human tissues, stem cell dynamics, single cell mutation burden, variant allele frequency, sampling, evolutionary inferences, Human

## Abstract

A new mathematical model that can be applied to both single-cell and bulk DNA sequencing data sheds light on the processes governing population dynamics in stem cells.

**Related research article** Moeller ME, Mon Père NV, Werner B, Huang W. 2024. Measures of genetic diversification in somatic tissues at bulk and single-cell resolution. *eLife* **12**:RP89780. doi: 10.7554/eLife.89780.

Various reservoirs of stem cells exist across the adult human body to ensure the production of certain populations of somatic cells. For instance, hematopoietic stem cells (HSCs for short) in the bone marrow continuously create the various types of blood cells that our body needs to carry oxygen, heal or defend itself. Simultaneously, these stem cells must be able to self-renew and increase their pool.

To perform these roles, stem cells rely on two types of division: symmetric and asymmetric. In an asymmetric division, a stem cell gives rise to one daughter cell that will differentiate into a somatic cell through further divisions, and one cell that retains stemness and ensures self-renewal. In a symmetric division, a stem cell generates either two differentiated cells or two stem cells.

Mutations accumulate within the genome of cells over time and successive divisions. These changes emerge due to biological processes such as errors in DNA replication or imperfect repair of genetic damage. The average frequency at which genetic sequences accrue mutations is known as the effective mutation rate.

The acquisition of these DNA changes results in tissues made up of cells with varied genetic information – an effect known as somatic heterogeneity – which can create significant diversity in the phenotypes of an organism. Evolutionary pressures which favor or hinder certain genetic variations also help to define these populations. However, these changes may result in the expansion of malignant cells or other harmful health effects. Clonal hematopoiesis, for example, is an age-related condition whereby a mutated HSC gives rise to a genetically distinct subpopulation of blood cells, and it is associated with higher risks of overt hematologic malignancies (Jaiswal and Ebert, 2019).

Understanding the dynamics of stem cell divisions can give scientists access to a range of crucial information, such as the number of stem cells in a tissue over time, their mutation rate or the frequency at which they engage in different types of division. Traditionally, capturing these processes has relied on lab-based methods such as visualizing cells through flow cytometry, cell barcodes analysis and immunofluorescence. In recent years, however, computational approaches have increased the knowledge of stem cell dynamics while also benefitting the clinical application of stem cells (see Pedersen et al., 2023a for a review of the importance of modelling for HSC dynamics; and Waters et al., 2021 for a review of how quantitative modelling of stem cell growth can impact regenerative medicine research). For instance, mathematical models have provided insights into poorly understood parts of the hematopoietic process in health and disease (Pedersen et al., 2023b; Ashcroft et al., 2017), including the simulation of how healthy and malignant HSCs compete under various conditions (Stiehl et al., 2020). They have helped to reconcile contradictory interpretations from different in vivo flux experiments (Takahashi et al., 2021), and to determine which factors may contribute to the successful transplantation of hematopoietic stem cells (Nakaoka and Aihara, 2012).

Sophisticated models have also been able to reconstruct the ‘phylogenetic tree’ of HSCs, as well as estimate the size of this population and how it changes through life (Lee-Six et al., 2018). These types of mathematical models rely on the fact that mutations accumulate over time per each division, and they have been applied to genome data collected from either single-cell or bulk DNA sequencing, with each level of resolution providing different information and being constrained by specific limitations. Now, in eLife, Marius Moeller, Nathaniel Mon Père, Weini Huang and Benjamin Werner report having developed a model that can capture key parameters of stem cell dynamics from both bulk and single-cell data, and shed light on somatic evolution (Moeller et al., 2024).

The team (who are based at Queen Mary University of London and institutes in Belgium and China) started by establishing a theoretical model of how mutations would accumulate through life in a healthy HSC population; this was based on cells dividing asymmetrically and symmetrically at different rates, and with spontaneous mutations taking place at each division. Three developmental stages were included: (i) an early phase during which the number of HSCs rapidly expands from a single cell through symmetric divisions; (ii) a maintenance phase where the overall population grows at a steady rate while also undergoing turnover via asymmetric divisions; and (iii) a final phase during which cells continue to divide asymmetrically but population numbers plateau (Figure 1).

**Figure 1.**
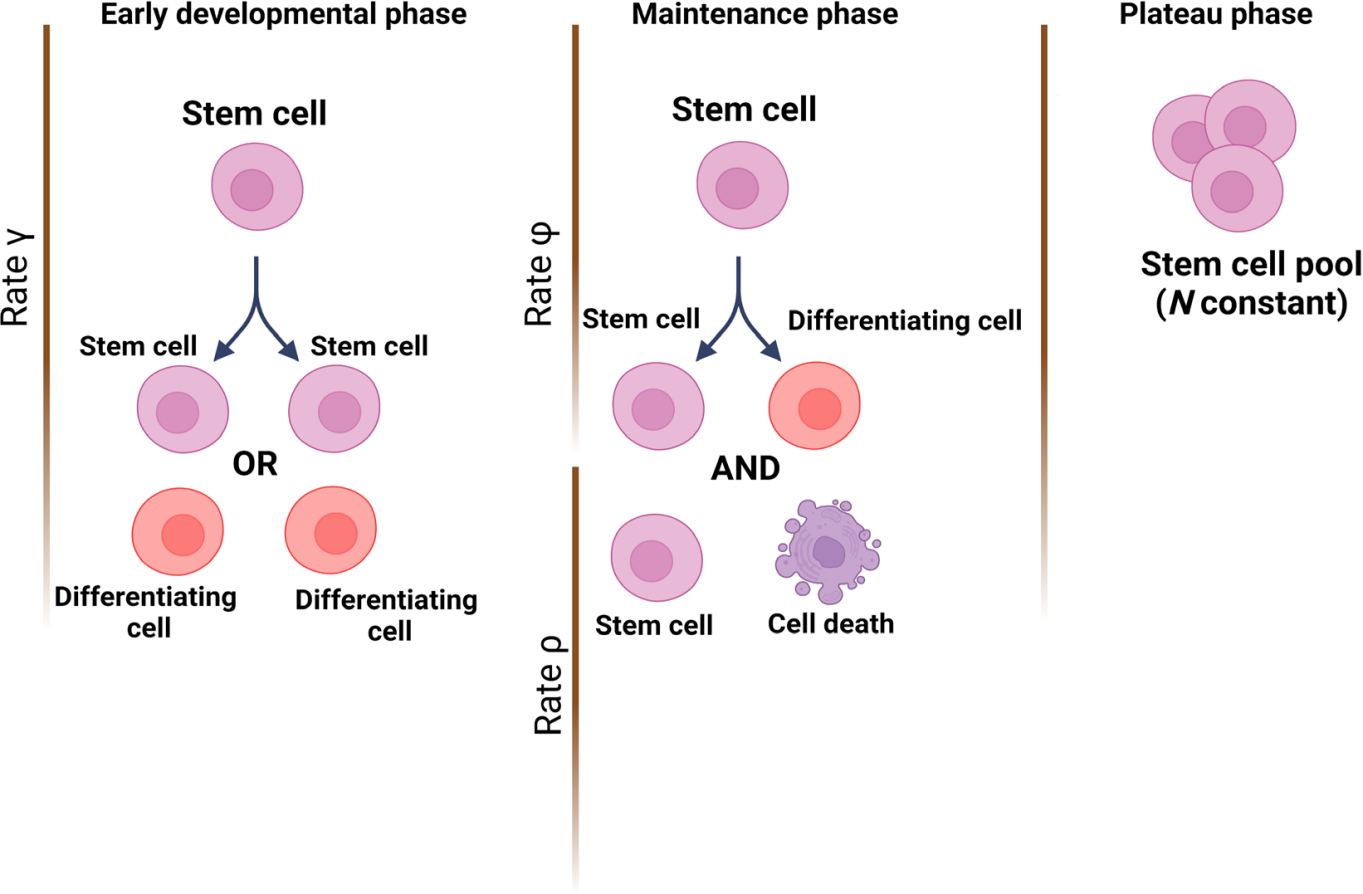
Modelling stem cell dynamics across development. The stochastic model designed by Moeller et al. establishes three phases, with each phase quantifying the number of stem cells and the dynamics of growth and/or removal due to differentiation or cell death. In the early developmental phase (left), the population grows rapidly due to stem cells engaging principally in symmetrical divisions (rate of divisions is represented as γ) to create either two stem cells (pink) or two cells that will differentiate into cells of the somatic tissue (red). In the maintenance phase (middle), the population grows at a slower pace, which includes ensuring the replacement of dead stem cells (rate ρ) and self-renewal via asymmetrical divisions (rate φ). In the plateau phase (right), the population size remains constant.

Next, Moeller et al. applied this model to bulk sequencing data from healthy oesophagus stem cells collected from individuals of various ages. The simulations suggested that the estimated effective mutation rate increased linearly with age. This could be interpreted as older cells having a higher mutation rate than younger ones; if so, this would lead to the total number of mutations in a cell increasing at a faster pace with age, which is known not to be the case. Instead, the team proposes that this result reflects the stem cell population slowly and linearly expanding in size with age, which upon sampling could mask as an increased mutation rate.

As bulk sequencing can only provide an average estimate of cell divisions and effective mutation rates, Moeller et al. then turned to single-cell data from HSCs obtained from one volunteer. While acknowledging the limitations inherent to working with relatively low cell numbers, they showed that their model was able to extract important population-level parameters from such a dataset, potentially allowing for qualitative analysis based on single-cell data. For instance, they could infer the proportion of asymmetric divisions in the HSC pool, as well as the maximal size of the population.

Based on this dataset, the model also provided an estimated effective mutation rate which was higher than expected based on the current understanding of the mechanisms that create random mutations. This led the team to suggest that existing models of somatic evolution may be incomplete, with biological processes which are not currently accounted for likely participating in mutation generation.

By coupling mathematic modelling with distinct aspects of genome sequencing technologies, the work by Moeller et al. offers an important examination of how mutations accumulate in somatic stem cells, like HSCs. As the team points out, it remains to be seen how other processes beyond mutation accumulation also help shape somatic heterogeneity throughout development, such as the effects of positive and neutral selection in young versus old age.
